# Effectiveness of a Nursing Intervention to Diminish Preoperative Anxiety in Patients Programmed for Knee Replacement Surgery: Preventive Controlled and Randomized Clinical Trial

**DOI:** 10.17533/udea.iee.v37n2e07

**Published:** 2019-09-19

**Authors:** Mauricio Medina-Garzón

**Affiliations:** 1 Nurse, Masters. Professor, Universidad Nacional de Colombia- Bogotá, Colombia. Email: mamedinaga@unal.edu.co Universidad Nacional de Colombia Universidad Nacional de Colombia Bogotá Colombia mamedinaga@unal.edu.co

**Keywords:** anxiety, arthroplasty, replacement, knee, control groups, motivational interview, orthopedics, perioperative nursing., ansiedade, artroplastia do joelho, grupos controle, entrevista motivacional, ortopedia, enfermagem perioperatória

## Abstract

**Objective.:**

This work was conducted to determine the effectiveness of a nursing intervention, based on the motivational interview, to diminish preoperative anxiety in patients programmed for knee replacement surgery.

**Methods.:**

Preventive type controlled and randomized clinical trial, on a sample of 56 patients programmed for knee replacement surgery in a clinic in Girardot (Colombia). Random assignment was made: an intervention group (*n=*28) and a control group (*n=*28). The six-question Amsterdam Preoperative Anxiety and Information Scale was applied before and after the intervention. The scale has a total score ranging from 5 to 30; the higher the score, the greater the preoperative anxiety. The nursing intervention was conducted in three sessions of motivational interview each lasting 40 min, during the six weeks prior to the surgical procedure; the control group received conventional management of education in the health institution.

**Results.:**

The mean score of preoperative anxiety was equal in the pre-intervention evaluation in both groups (19.76 in the experimental versus 22.02 in the control =22.02; *p*<0.226), while during the post-intervention, the anxiety score was lower in the intervention group compared with the control group (15.56 and 20.30, respectively; *p* <0.013).

**Conclusion.:**

Nursing intervention based on the motivational interview was effective in diminishing preoperative anxiety in patients programmed for knee replacement surgery.

## Resumo

**Objetivo.:**

Determinar a efetividade de uma intervenção de enfermagem baseada na entrevista motivacional, para diminuir a ansiedade pré-operatória em pacientes programados para cirurgia de prótese de joelho.

**Métodos.:**

Ensaio clínico controlado e aleatorizado tipo preventivo, numa amostra de 56 pacientes programados para cirurgia de prótese de joelho numa clínica em Girardot (Colômbia). Foram designados em forma aleatória: um grupo de intervenção (*n*=28) e um grupo de controle (*n*=28). Se aplicou antes e depois da intervenção, a escala de seis perguntas, APAIS (The Amsterdam Preoperative Anxiety and Information Scale), a qual tem uma pontuação total que vá de 5 a 30 a mais pontuação, é maior a ansiedade pré-operatório. A intervenção de enfermagem se desenvolvimento em 3 sessões de entrevista motivacional com uma duração de 40 minutos, durante as 6 semanas anteriores à realização do procedimento cirúrgico; o grupo de controle recebeu o manejo convencional de educação na instituição de saúde.

**Resultados.:**

A média da pontuação de ansiedade pré-operatória foi igual na avaliação pré-intervenção nos dois grupos (19.76 no experimental versus 22.02 no controle = 22.02; *p*<0.226), enquanto que na pós-intervenção a pontuação de ansiedade foi menor no grupo de intervenção comparado com o grupo controle (15.56 e 20.30, respectivamente; *p*<0.013).

**Conclusão.:**

A intervenção de enfermagem baseada na entrevista motivacional foi efetiva na diminuição da ansiedade pré-operatória em pacientes programados para cirurgia de prótese de joelho.

## Introduction

Progress in the nursing profession regarding caring for people within surgical services is evident; clear examples of this are noted in the safety policy for surgical patients,(1) good sterilization and instrumentation practices in the operating room, and nursing interventions in favor of patient safety and decreased risks associated to anesthesia and surgery.(2) However, in spite of these transcendental breakthroughs, work has not been sufficient on the emotional responses of patients upon a surgical procedure. This type of event becomes a powerful stressor of complex nature for the individual, given that the process depends not only on the hospitalization, but also on the events and consequences generated on the environment prior, during, and after surgery.(3) The surgical environment causes fear and anxiety regarding the surgery or the hospital stay.(4) All these responses can produce side effects to the treatment and possible isolation of patients in their social and family setting, subsequently generating physical and psychological affectations to the point of being able to impact upon a chronic disease. associated with perioperative complications, or even due to the socioeconomic impact by increasing post-surgery morbidity.(5) In this respect, emotional responses of surgical patients have been reported, like anxiety by 72%, fear by 68.5%, and tension by 59.0%, which implies the impact it may have upon a person when entering the surgical room.(6) 

Specifically, anxiety emerges weeks prior to the surgical event and symptoms intensify during the hours prior to admission, which produces physiological and emotional effects upon surgery, a fundamental reason to provide preoperative information before patients enter the operating room. Upon addressing the phenomenon of preoperative anxiety in surgical patients, it is evident that such persist up to 12 h during post-surgery and is accompanied by manifestations, like: high blood pressure, diaphoresis, or headache (in spite of having had initial treatment with anxiolytics), which can delay recovery and prolong the hospital stay.(7) In spite of this impact generated by anxiety, literature shows that from nursing, strategies have been implemented to diminish it in this context, providing verbal information of the surgical procedure or by using pre-surgical kits in elective surgeries (like, inguinal or umbilical hernias, tonsillectomy, adenoidectomy or circumcision).(8) Other relaxation techniques implemented for surgical procedures are reported in studies with surgery patients due to breast cancer, patients with fibroadenoma or with fibrocystic disease, achieving decreased anxiety in many cases.(9)

Globally, the number of surgical procedures has increased. According to the World Health Organization,(10) annually around the world over 4-million patients are subjected to surgery and it is estimated that 50% to 75% develop some degree of anxiety during the preoperative period. In the same sense, anxiety is considered a public health problem, given that it affects 10% of the global population, hindering the work of the health staff in the recovery of the subject, with regards to the treatment guidelines employed.(11) The aforementioned manifests the risk patients are exposed to and the need to diminish it through prevention strategies. Specifically, the person experiences more anxiety just before the surgery, especially while awaiting for the intervention and in part due to the circumstances surrounding this event.(12) Due to this, it is proposed that the person who will be surgically intervened must have the motivational interview during the preoperative period, a concept developed by Miller and Rollnick.(13) It consists in a type of patient-focused clinical interview, whose purpose is that of exploring and solving ambivalences about a behavior or unhealthy habit to, thus, promote changes toward healthier lifestyles. The motivational interview enables patient positioning toward the desire for change and helps them to recognize and care for their current and future problems enhancing their perception of self-efficacy.(14) To conduct it, it is indispensable to identify the patient’s motivational stage to propose and develop different actions that potentiate motivation upon the behavioral change.(15) 

From this vantage point, the role of nursing in participating in the preoperative assessment is fundamental when exploring anxiety, as of the motivational interview. Because of the aforementioned, research was conducted to determine the effectiveness of a nursing intervention based on the motivational interview in diminishing preoperative anxiety in individuals programmed for knee replacement surgery. 

## Methods

Study design. This was a preventive type controlled randomized clinical trial.

Participants. The population involved individuals programmed for knee replacement surgery in a tier III specialized clinic in the city of Girardot (Colombia), who were admitted to preoperative assessment, during the period comprised between January 10 and April 30, 2018. The inclusion criteria included people ranging in age between 50 and 75 years, patients programmed in the institution for knee replacement under two months from the procedure, and the patients’ acceptance to participate in the study. The exclusion criteria were: individuals with intellectual cognitive disability (behavioral-cognitive intervention), individuals programmed for a surgical procedure different to arthroplasty or knee replacement, and the patient’s refusal to participate in the research. This trial is inscribed in the Brazilian registry of clinical trials (REBEC, for the term in Portuguese): Trial: (Req: 7545, Effectiveness of a Nursing Intervention to Reduce Preoperative Anxiety). 

Intervention. To carry out the intervention, participants from the experimental and control groups received an individual and informative session by nursing on the surgical preparation and the procedure. On the first visit, a deliberate questionnaire was applied, to identify the patient, general characteristics, and verify compliance with the criteria. The control group received the habitual treatment, while the experimental group received, besides the habitual, the motivational interview. Prior to performing the trial, both groups were applied the Amsterdam Preoperative Anxiety and Information Scale (APAIS), which is based on a six-item questionnaire (I am worried about the anesthetic; The anesthetic is on my mind continually; I would like to know as much as possible about the anesthetic; I am worried about the procedure; The procedure is on my mind continually; and I would like to know as much as possible about the procedure), with response options evaluated in a Likert-type scale from 1 to 5; one meaning not at all and 5 extremely. The first two relate to anxiety due to the anesthetic, numbers four and five relate to anxiety due to the surgery and the sum is considered as preoperative anxiety that can vary from 5 to 30 points. The APAIS has validity and reliability with a Cronbach’s alpha internal consistency of 0.84 .(16) The APAIS was applied by two nursing professionals, as evaluators, at the start and end of the procedure. Three sessions of the motivational interview were conducted within a 20-day period and, thereafter, the follow up took place four weeks later. The motivational interview sessions are mainly based on participants establishing their own goals to slowly change their lifestyles. Each session lasted approximately 40 min, which began by exploring their level of anxiety and the triggering factors during the eight days prior to the interview. 

Sample size. A sample of 56 subjects was calculated, distributed randomly (*n =* 28) for the intervention group and (*n =* 28) for the control group, bearing in mind the inclusion criteria and 95% CI, power of 80%, margin of error ± 4.4%, and a maximum proportion of 52.4% of patients in the experimental group who would improve anxiety compared to 47.6% in the control group. Patient selection was carried out randomly by sequence generation, using a list of numbers registered in the database according to the numerical codes assigned by the researcher upon entering the study. The final sample included 55 patients: 28 in the intervention group and 27 in the control group because of the voluntary withdrawal of one patient in this last group. 

Assignment reservation. Patients complying with the inclusion criteria were scheduled in the consultation service. The researcher proceeded to make the assignment through identification codes of the participants. Thereafter, the participants were assigned to each group, using a computer-generated random table. To hide the assignment, it was serially numbered, keeping a printed copy of the software-generated sequence. Contamination of the study was controlled by guaranteeing that it was conducted in personalized manner in different sites and days, to keep participants from sharing the information with each other.

Masking. This was a double-blind study in which participants ignored to which group they had been assigned and the professional nurses, considered evaluators, ignored the selection of patients and the random assignment. In addition, they did not participate in the intervention.

Data analysis. A prior descriptive analysis was conducted to identify differences in the general characteristics of the groups studied; Student’s t test was used for means of the quantitative variables and the Chi squared for proportions. To evaluate changes in the APAIS scores between the initial and final moments intra-subject, and study intergroup used ANOVA with repeated measures. Mauchly’s W was used to assess if the variances-covariances matrix was spherical. When the assumption of sphericity was fulfilled, the F test was used, which indicated whether to accept or reject the hypothesis of equality between the study groups in the APAIS score during both assessment moments. The contrast used in this procedure is of polynomial type to the factors of repeated measures, which permitted studying the relationship between the factor (study group) and dependent variable (APAIS score) is linear. 

Ethical aspects. This research was approved by the Ethics Committee at Universidad Nacional de Colombia and the research committee of the institution where the study took place. Previo a su participación se solicitó de manera voluntaria and por escrito, el consentimiento informado a los participantes en el estudio and la confidencialidad de la información.

## Results

**Figure 1:**
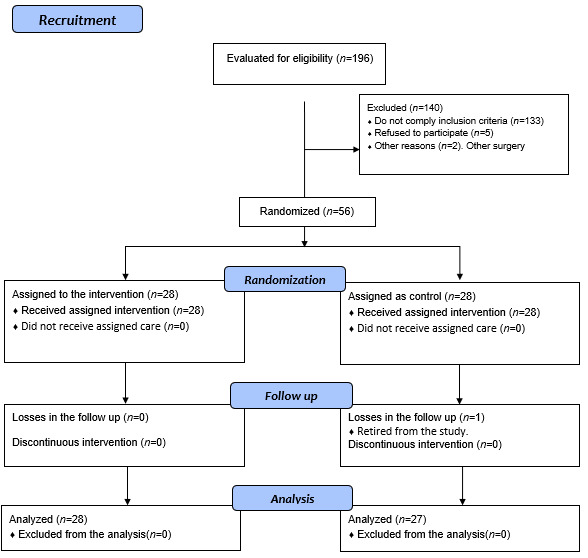
Study flow diagram

This study recruited 196 patients of which 140 were excluded because they did not comply with the requirement of knee replacement surgery and 133 did not comply with the inclusion criteria (Figure 1); for a total of 56 participants, distributed 28 in each group (experimental and control). One of the participants in the control group was excluded due to voluntarily withdrawing from the study during the second week of the nursing intervention. At the end of the study, the sample comprised 28 patients in the intervention group and 27 patients in the control group.

Table 1 shows that no statistically significant differences existed between the study groups in relation to the sociodemographic and clinical characteristics. Generally, it can be said that the participants almost have a ratio of one man per woman, ranging in age between 73 and 76 years and most had family support. Regarding the clinical variables, the antecedent of prior surgeries prevailed among the patients, as well as non-consumption of anxiolytics, risk upon anesthesia was classified from 2 to 4 in the ASA scale; general anesthesia was used during the procedure. For the participants, the major cause of preoperative anxiety is the effects of the anesthetic.

**Table 1:** General characteristics of the study groups

Variable	Control group (n = 27)	Intervention group (n = 28)	p-value
Sex; n (%)			0.483
Man	15 (55.5)	14 (50.0)	
Woman	12 (44.4)	14 (50.0)	
Age; mean ±SD	73.7±16.6	76.32±16.1	0.366
ASA Classification; n (%)			0.082
0-1	9 (33.3)	6 (21.4)	
2-4	18 (66.6)	22 (78.6)	
Type of anesthesia; n (%)			0.536
General	27 (100)	28 (100)	
Other	0 (0)	0 (0)	
Prior surgeries; n (%)			0.064
No	12 (44.4)	7 (25)	
Yes	15 (55.5)	21 (75)	
Anxiolytics; n (%)			0.599
No	26 (96.3)	28 (100)	
Yes	1 (3.7)	0 (0)	
Social support; n (%)			0.537
Family	21 (77.8)	23 (82.1)	
Others	6 (22.2)	5 (17.9)	
Cause of the anxiety; n (%)			0.397
Anesthetic	18 (66.7)	17 (60.7)	
Procedure	4 (14.8)	8 (28.6)	
Complications	5 (18.5)	3 (10.7)	

Table 2 shows that, although both groups diminished the anxiety score over time, in the intervention group the difference between both assessment moments is of 4.2 points, while in the control group it is of 1.73 points. The mean score of preoperative anxiety in the post-intervention evaluation was 5 points lower in the intervention group, compared with the control group, with this difference being statistically significant. 

**Table 2:** Comparison of the total average score of preoperative anxiety in the study groups before and after the procedure

		Moment	Moment
Group	Measurement		
		Before	After
Intervention (n=28)	Mean ±SD	19.76±8.56	15.56±8.85
Intervention (n=28)	IC95% of the mean	16.37-23.14	12.12-18.99
	Mean ±SD	22.02±9.35	20.30±8.19
Control (n=27)	95%CI of the mean	18.39-25.64	17.06-23.54
	Difference of means between groups	2. 26	5.04
	Bilateral p-value	0.226	0.013

Analysis of repeated measures. In this study, 28 patients from the intervention group and 27 patients from the control group completed both APAIS assessments. In the repeated measures ANOVA, Mauchly’s W was 1.0, assuming sphericity and the F test was used (F=14.43, *p*<0.001) that indicated linear relation between the APAIS score and the study group. The size of the effect was 0.214. In addition, the multivariate model showed a global difference of 3.1±0.36 points (95%CI: 2.36-3.84) between both study groups.

## Discussion

This study on the effect of a nursing intervention based on the motivational interview to diminish preoperative anxiety in patients programmed for knee replacement surgery revealed that after six weeks of follow up, the preoperative anxiety score was lower in the group receiving the intervention compared with the control group. The results are consistent with those obtained by Rojas *et al.,*(17) which implemented a nursing educational strategy to diminish anxiety in patients during the pre-operative and post-operative; although anxiety was measured with the Beck test. 

In spite of progress in nursing interventions, anxiety remains a problem in patients. However, nursing has revealed that visits during the preoperative stage reduce anxiety and post-surgery complications in patients programmed for laparoscopy surgery.(18) Likewise, Amini *et al.*,(19) through a randomized clinical trial, compared the effect of using brochures plus verbal advice for education about preoperative anxiety and used Spielberger’s Trait/State Anxiety Inventory before and after the intervention. They found significant difference between the mean scores of the state anxiety scale between the intervention groups (brochure and verbal) with the control group, additionally recommending the use of well-designed brochures. Another randomized clinical trial in patients programmed for herniated disc surgery used the multimedia strategy to educate about anxiety during nursing visits, finding statistically significant difference between both groups in terms of preoperative anxiety, besides improving vital signs.(20) 

Due to the aforementioned, studies recognize the importance of the communication skills nurses must have to approach and care for patients upon a surgical event, which can take place through assessment and follow up by implementing the motivational interview, considered an effective way of improving attitudes and behaviors in individuals by using persuasion and trust.(21) However, another study(22) states that the conventional informative intervention used by nurses and the rest of the health staff does not diminish anxiety in patients. Jiménez *et al*.,(23) found that patients with osteoarthritis of knees feel commonly anxious and that after surgery the level of anxiety drops, but that this change is also related with the degree of satisfaction with the procedure. Another aspect also related with reduced anxiety is that of maintaining empathic and collaborative communication with patients, through informative and persuasive intervention.(24) Due to this, this study adds to existing evidence that interventions based on motivational techniques have resulted effective in other groups, permitting acceptance of the surgery and subsequent changes in lifestyle to comply with recommendations and, finally, improving adherence to the treatment.(25)

To conclude, the nursing intervention based on the motivational interview was effective in diminishing preoperative anxiety in patients programmed for knee replacement surgery. Follow up of patients programmed for orthopedic surgery is of vital importance for nurses to recognize situations and circumstances that cause them anxiety, with the purpose of conducting clinical and informative advice and enhancing care during all the stages of the surgical process. Studies are recommended to investigate the effect of nursing interventions in surgical patients and other related areas. The limitation of the investigation was the availability of a single researcher and, therefore, selection bias cannot be ruled out by the sampling. 
